# The RelB alternative NF-kappaB subunit promotes autophagy in 22Rv1 prostate cancer cells *in vitro* and affects mouse xenograft tumor growth *in vivo*

**DOI:** 10.1186/1475-2867-14-67

**Published:** 2014-07-28

**Authors:** Ingrid Labouba, Alexis Poisson, Julie Lafontaine, Nathalie Delvoye, Philippe O Gannon, Cécile Le Page, Fred Saad, Anne-Marie Mes-Masson

**Affiliations:** 1 Centre de recherche du Centre hospitalier de l’Université de Montréal (CRCHUM)/Institut du cancer de Montréal, Montreal, Canada; 2 Division of Urology, CHUM, Université de Montréal, CHUM Notre-Dame, 1560 Sherbrooke east, Montreal, Quebec, Canada; 3 Department of Medicine, Université de Montréal, Montreal, Canada

**Keywords:** RelB, Tumor initiation, Proliferation, Anchorage-independent cell growth

## Abstract

**Background:**

The involvement of NF-κB signaling in prostate cancer (PCa) has largely been established through the study of the classical p65 subunit. Nuclear localization of p65 in PCa patient tissues has been shown to correlate with biochemical recurrence, while *in vitro* studies have demonstrated that the classical NF-κB signaling pathway promotes PCa progression and metastatic potential. More recently, the nuclear location of RelB, a member of the alternative NF-κB signaling, has also been shown to correlate with the Gleason score. The current study aims to clarify the role of alternative NF-κB in PCa cells by exploring, *in vitro* and *in vivo,* the effects of RelB overexpression on PCa biology.

**Methods:**

Using a lentivirus-expression system, we constitutively overexpressed RelB or control GFP into 22Rv1 cells and monitored alternative transcriptional NF-κB activity. *In vivo*, tumor growth was assessed after the injection of 22Rv1-derived cells into SCID mice. *In vitro*, the impact of RelB on 22Rv1 cell proliferation was evaluated in monolayer culture. The anchorage-independent cell growth of derived-22Rv1 cells was assessed by soft agar assay. Apoptosis and autophagy were evaluated by Western blot analysis in 22Rv1-derived cells cultured in suspension using poly-HEMA pre-coated dishes.

**Results:**

The overexpression of RelB in 22Rv1 cells induced the constitutive activation of the alternative NF-κB pathway. *In vivo*, RelB expression caused a lag in the initiation of 22Rv1-induced tumors in SCID mice. *In vitro*, RelB stimulated the proliferation of 22Rv1 cells and reduced their ability to grow in soft agar. These observations may be reconciled by our findings that, when cultured in suspension on poly-HEMA pre-coated dishes, 22Rv1 cells expressing RelB were more susceptible to cell death, and more specifically to autophagy controlled death.

**Conclusions:**

This study highlights a role of the alternative NF-κB pathway in proliferation and the controlled autophagy. Thus, the interplay of these properties may contribute to tumor survival in stress conditions while promoting PCa cells growth contributing to the overall tumorigenicity of these cells.

## Introduction

Prostate cancer (PCa) is the most frequently diagnosed cancer and the second cause of cancer-related death in men in the United States [1]. Advanced or recurrent PCa are usually treated with androgen deprivation therapy (ADT). While the majority of patients will initially respond, a significant proportion will eventually become refractory to ADT. This castration resistant state of the disease is ultimately fatal. Despite numerous reported studies, there remains many unanswered questions regarding biological mechanisms of PCa progression and the parameters to predict disease progression [2]. Based on previous work reported by our group, and supported by others, nuclear factor kappa B (NF-κB) appears to be a central molecular player in PCa progression and may represent a potential prognostic biomarker [3-9].

NF-κB is a family of transcription factors encompassing structurally related proteins characterized by their Rel-homology domain and that have to form dimers to be functional. The RelA (p65), RelB and c-Rel proteins carry a transactivation domain. The p105 and p100 subunits are characterized by an ankyrin-repeat domain whose cleavage produces the p50 and p52 subunits, respectively. NF-κB subunits dimers are retained inactive in the cytoplasm due to the binding with inhibitory proteins IκB. Upon cell stimulation, the IκB undergo proteosomal degradation and NF-κB dimers translocate into the nucleus to transactivate their target genes. NF-κB transduces its signal through two major pathways: the classical and the alternative. Activation of the classical NF-κB pathway involves the proteasomal degradation of IκB protein and the release of p65/p50 dimer. In the alternative pathway, p100 acts as an IκB-like protein by retaining RelB/p100 dimer in the cytoplasm. The activation signal leads to p100 phosphorylation and its partial proteasomal degradation, thereby producing RelB/p52 dimers [10,11]. The cellular responses leading to the activation of either the classical or the alternative pathway depend on a variety of cytokines and cellular stress responses. While several receptors can initiate classical NF-κB signaling, only TNFRSF (Tumor necrosis factor-receptor super-family) can induce the alternative pathway activation [12].

While the classical pathway has been extensively studied and its involvement in tumorigenesis is well established, the alternative pathway has been less extensively studied. Studies have nonetheless associated the alternative NF-κB pathway with increased tumorigenic potential. For instance, data from our group suggest that the nuclear distribution of the alternative NF-κB subunits RelB and p100/p52 in tumor tissues of PCa patients correlates with an activation of the alternative NF-κB pathway and potentially involved in PCa progression [13]. Moreover, RelB expression in LNCaP and PC3 PCa cell lines increase their ability to form tumors in a mouse model through the modulation of IL-8 and PSA expression [14,15]. In breast cancer, RelB stimulates cell proliferation [16], invasion [17] and increases resistance to anti-cancer therapies [18,19]. While our understanding of the alternative NF-κB pathway is growing, the cell mechanisms impacted by the alternative NF-κB pathway within cancer cells remains to be further explored.

Here, we used the 22Rv1 PCa cell line to explore the role of RelB expression and the alternative NF-κB pathway on cell functions. We derived 22Rv1 cell populations overexpressing RelB leading to a constitutively active alternative NF-κB pathway. We found that RelB expression caused a lag in 22Rv1 cells tumor initiation, although overall tumor growth in SCID mice was not affected. Additional *in vitro* functional assays revealed that RelB reduced anchorage-independent cell growth in soft agar, but increased the proliferative potential of 22Rv1 cells in adherent conditions. We also demonstrated that RelB appeared to sensitize 22Rv1 cells to autophagy. This is the first report to suggest a regulatory effect of the alternative NF-κB pathway on autophagy. The integration of our *in vitro* and *in vivo* results lead us to propose a model of RelB function during tumor initiation and progression in the xenograft mouse model.

## Material and methods

### Cell line and culture conditions

22Rv1 human prostate carcinoma epithelial cells were obtained from ATCC and cultured in RPMI-1640 complete media (Wisent, Montreal, Qc) containing 10% FBS (Fetal Bovine Serum) (Wisent, Montreal, Qc), 2.5 μg/mL amphotericin B and 50 μg/mL gentamicin (Gibco, Grand Island, NY), at 37°C with 5% CO_2_. The 22Rv1 derivatives cells expressing GFP or RelB were grown under selection in RPMI-1640 complete media supplemented with 1.5 μg/mL of puromycin (Sigma, St. Louis, MO).

### Lentiviral production and transduction

RelB (NM_006509, from OriGene, Rockville, MD, USA) was inserted in pENTR/D-TOPO (Invitrogen, NY, USA). The generated pENTR-RelB vector was recombined in the 670–1 vector (pLenti CMV/TO Puro DEST, Addgene 17293) [20] using recombination-cloning technology from Invitrogen. The eGFP was used for control cell population and has previously been described elsewhere [21,22]. Lentiviruses were produced by co-transfecting vectors containing RelB or eGFP cDNA and using the ViraPower Lentiviral Packaging Mix (Invitrogen, Carlsbad, CA) in the 293FT packaging cell line. The lentiviral contructs were harvested from cell supernatants, concentrated by ultracentrifugation (20,000 rpm) and stored at −80˚C until use. For viral infection, cells were plated in 6-well plates containing 2 ml of culture media and cultured until 50-70% confluence. Infections were performed in RPMI 1640 media containing 5 μg/ml polybrene (Sigma, St. Louis, MO). Culture media was changed 16 hrs after the infection and puromycin selection was performed two days post-infection.

### Xenograft tumor assays

Six week old male SCID CB17 mice (Charles River, Montreal, QC, Canada) were injected subcutaneously with 2.5 × 10^5^ cells resuspended in a mix of 1:1 1X PBS and matrigel (BD Biosciences, Mississauga, ON, Canada). Six mice were used for each experimental group. Controls included one group of mice injected with a mixed population of 22Rv1-GFP cells and another with a clonal population of 22Rv1-GFP cells. Three other experimental groups were injected with three independent 22Rv1-RelB clonal populations. Data on the weight of the mice and dimensions of the tumors were collected twice a week. Mice were housed under sterile conditions during all experimentations and were sacrificed when neoplastic lesions reached the limit point (2500 mm^3^) established by the Institutional Committee on Animal Protection (ICAP) according to the Canadian Council on Animal Care (CCAC). The tumors were then harvested, fixed in formalin and embedded in paraffin (FFPE tissues) for subsequent histological analyses.

### Immunohistochemistry

The 22Rv1-induced tumors and 22Rv1 cells were stained by immunochemistry to monitor RelB expression, as previously described by our group [23]. *In vitro*, cells were cultured directly on sterile slides to reach 70-80% of confluence. After two washes with 1X PBS, they were fixed for 20 min in formalin (ACP Chemicals Inc., Montreal, QC, Canada) before a 15 min blocking step with serum-free blocking reagent. Subsequent steps were the same for FFPE tissues and cells samples.

Anti-RelB antibody (C-19, Santa Cruz Biotechnology Inc., Santa-Cruz, CA, USA) was used at a dilution 1:500 (FFPE tissue samples) or 1:750 (cell samples) in 1X PBS. Substitution of the primary antibody with 1X PBS served as a negative control.

### Protein extraction and immunoblotting analyses

For total protein extracts, cells were lysed with cold lysis buffer for 30 min on ice [10 mM Tris–HCl pH 7.4, 150 mM NaCl, 1 mM EDTA pH 8.0, 1 mM DTT, 1 mM NaF, 0.5% NP-40, 10 mM sodium orthovanadate/complete protease inhibitor cocktail (Roche Diagnostics, Laval, QC, Canada)]. The cytoplasmic/nuclear extracts were prepared as previously described by our group [24]. Protein extracts were analyzed by Western blot using SDS-polyacrylamide gels (10 or 12.5%) and transferred onto nitrocellulose membranes (Biorad), and signal was revealed using ECL (GE Healthcare, Piscataway, NJ, USA). Membranes were probed with anti-β-Actin antibodies as a loading control. GAPDH was used as a purity indicator for nuclear extracts. The antibodies used for protein expression analyses were: anti-RelA (sc-8008), anti-RelB (sc-226), anti-PARP-1/2 (sc-7150) antibodies (Santa Cruz Biotechnology Inc., Santa Cruz, CA); anti-p100/p52 (05–361) (Upstate Biotechnology, Charlottesville, VA); anti-LC3 (NB100-2220) (Novus biological, Oakville, ON); anti-GAPDH (AB9485-100) and anti-β-Actin (AB6276-100) (Abcam, San Francisco, CA).

### Immunoprecipitation of RelB

Protein samples were incubated with protein A/G agarose (sc-2003, Santa-Cruz Biotechnology Inc) and normal rabbit IgG (sc-2027, Santa-Cruz Biotechnology.inc) for a pre-clearing step. *ImmunoCruz™ IP/WB Optima F System* (sc-45043, Santa-Cruz Biotechnology Inc) was used for immunoprecipitations. The matrix (25 μL/sample) and anti-RelB (1 μg/sample) antibody were pre-incubated in 1X PBS for antibody/matrix complex formation required for subsequent steps. Pre-cleared protein samples (250 μg) were then incubated with 500 μL of matrix/anti-RelB complexes overnight at 4°C to precipitate RelB protein. The immunoprecipitated fraction was washed with cold lysis buffer (10 mM Tris–HCl, pH 7.4, 150 mM NaCl, 1 mM EDTA pH 8.0, 1 mM DTT/1 mM NaF/10 mM sodium orthovanadate/protease inhibitor cocktail). Immunoprecipitated proteins still associated with matrix were then denaturated prior to loading for Western blot analyses as described above.

### NF-κB gene reporter assay

The transcriptional activity of NF-κB was addressed using a Dual-Glo® Luciferase Assay System (Promega, Madison, WI). The p3enh-κb-CONAluc, carrying a *Firefly* luciferase gene downstream of the κB consensus sequence trimer, was used as previously described [25]. The phRL-CMV vector used as internal control contains the CMV promoter upstream of a synthetic *Renilla* luciferase gene (Promega, Madison, WI).

The 22Rv1 cell lines, either wild type (WT), RelB or GFP transduced, were co-transfected with p3enh-κb-CONAluc and phRL-CMV reporter vectors. The *Firefly* and *Renilla* luciferase luminescence were measured 48 hrs post-transfection. The relative NF-κB transcriptional activity was expressed according to the ratio of luciferase activity in the samples under study (RelB and GFP expressing cells) normalized against 22Rv1 WT control cells values. The experiment was repeated three times.

### Soft agar assay

The ability to grow in anchorage-independent conditions was assessed by culturing 22Rv1 cells in soft agar. Cells were trypsinized, suspended in 0.33% agar in RPMI 1640 complete media and plated in 6-well plates pre-coated with 0.66% agar. Cells were then incubated in soft agar for two weeks at 37°C and 5% CO_2_. Cell culture media was added weekly. The colonies were photographed and manually counted after coloration step with 0.1% crystal violet in 2% methanol. Three independent experiments in duplicate were performed.

### Cell growth assay

A total of 1 × 10^5^ cells were seeded onto 6-well plates at day 0. Starting at day 1, we counted the cells every 48 hrs until day 9 using the *CASY Model TT cell counting* device (Roche Innovatis AG, Basel, Switzerland). Each experiment was performed in duplicate and repeated three times.

### Suspension culture and cell death assay

Tissue culture plates were coated with poly-2-hydroxyethyl methacrylate (poly-HEMA) (Sigma, St. Louis, MO). The poly-HEMA solution (20 mg/mL) was dissolved at 65°C in 95% ethanol under stirring condition. Plates underwent two coating steps with poly-HEMA before use. After rinsing the wells with 1X PBS, 2×10^3^ (96-well plates for apoptosis assay) or 2×10^5^ (6-well plates for protein extraction) cells were plated and cultured for seven days. The cell death rate was determined using the CytoTOX-Glo bioluminescent cytotoxicity assay according to manufacturer instructions (Promega Inc, WI, USA). Bioluminescence was read using a *Wallac 1420 multilabel counter* (PerkinElmer, Turku, Finland).

### Statistical analysis

The Anova one-way followed by Tukey’s post-test was used for multiple comparisons, while Mann–Whitney U-test was used for comparison of one variable between two groups. The Kruskal-Wallis test was used for comparisons of multiple variables, at each time point for an experiment, and between different groups. All statistical tests were carried out using SPSS 16.0 software (SPSS Inc, Chicago, IL, USA). *P*-value < 0.05 was considered as statistically significant.

### Ethical approval

Six week old male SCID CB17 mice were purchased from Charles River Laboratories (Montreal, QC, Canada). and were maintained on site in the CHUM-Hopital Notre Dame animal facilities. All mouse experiments were performed strictly according to the Comité Institutionnel de la Protection des Animaux (CIPA) ethics guidelines and permissions.

## Results

### Exogenous RelB expression in 22Rv1 cells induced the activation of the alternative NF-κB pathway

**Figure 1:**
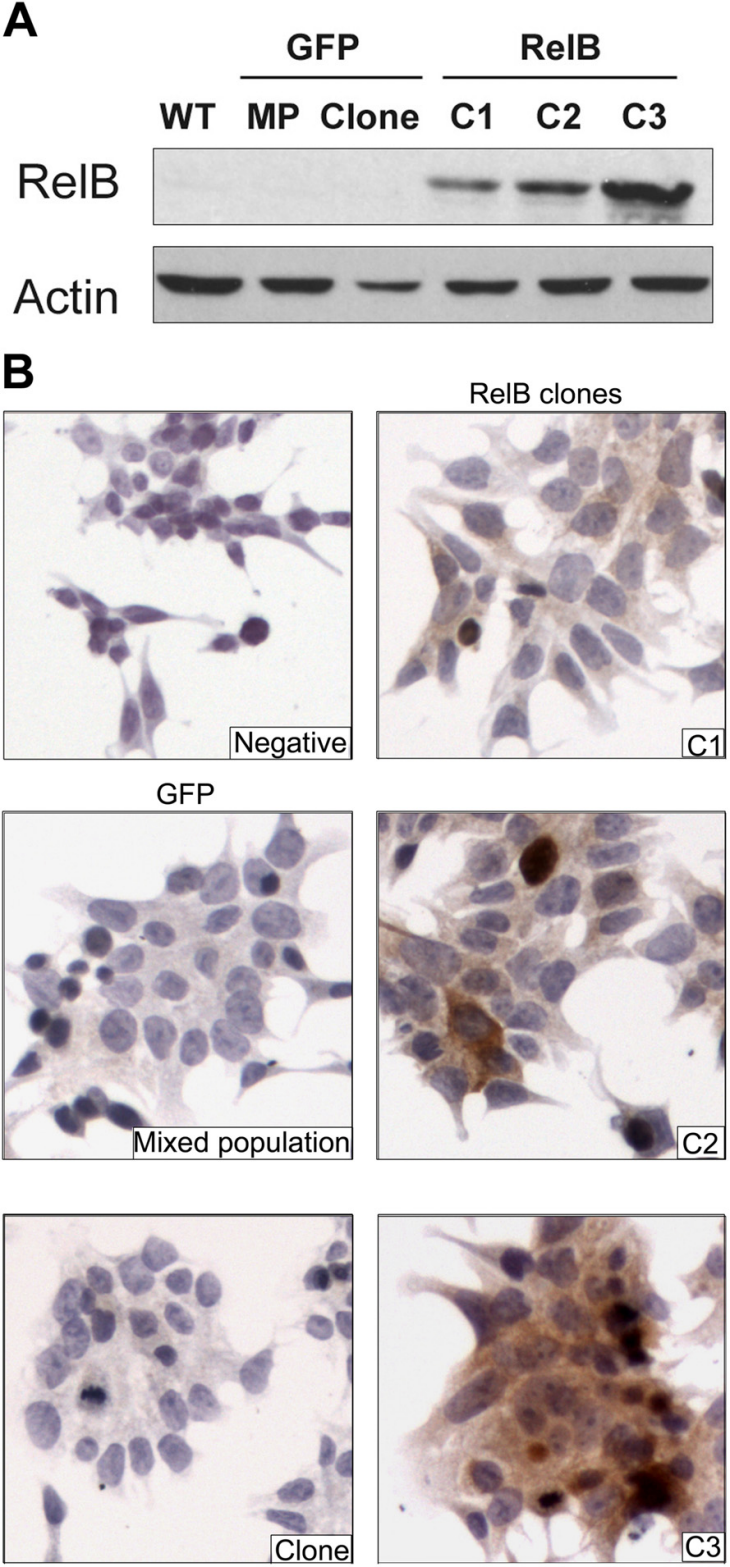
**RelB expression profile in 22Rv1-derived cells. A)** RelB immunoblotting from whole protein extracts of wild type (WT) and transduced (RelB, GFP) 22Rv1 cells. **B)** Immunocytochemical staining of RelB on 22Rv1-derived cells: RelB clones (C1, C2, and C3) and GFP control cells (mixed population and clone). In the negative control, 1X PBS replaced RelB antibody.

**Figure 2:**
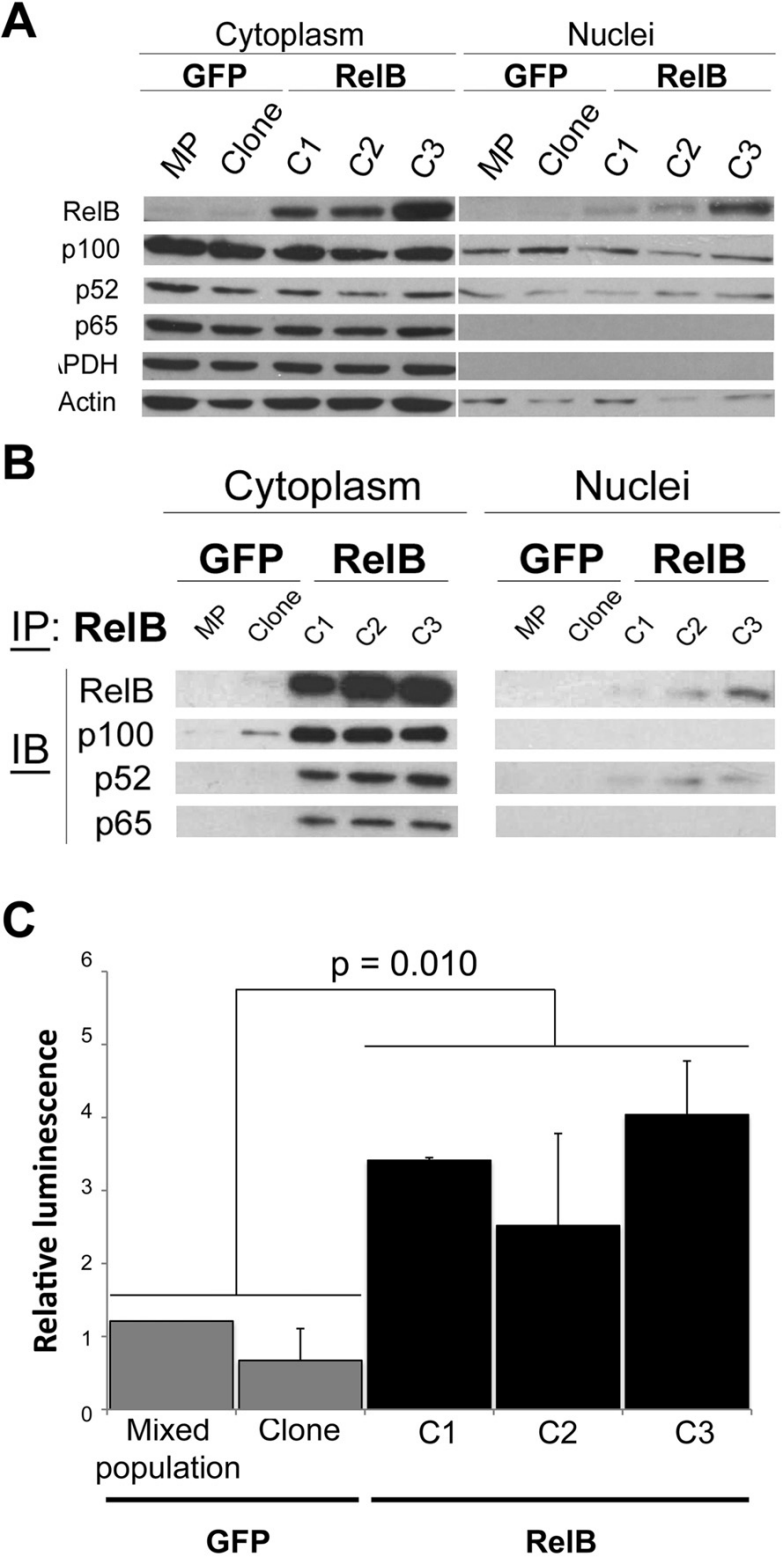
**RelB sub-cellular localization and alternative NF-κB activity in 22Rv1-derived cells. A)** Cellular distribution of the main NF-κB subunits analyzed by immunoblotting on protein extracts of cytoplasmic and nuclear compartments from RelB clones and GFP control cells. Actin was used as loading control for both cytoplasmic and nuclear protein extracts whereas GAPDH was used as a purity indicator for nuclear protein extracts. **B)** Immunoprecipitation of RelB from cytoplasmic and nuclear compartments protein extracts from RelB clones and GFP control cells. Immunoblot analyses of p100, p52 and p65 were performed from immunoprecipitated RelB fraction. **C)** NF-κB transcriptional activity by luciferase gene-reporter assay. RelB clones and GFP control cells were co-transfected with p3enh-κB-CONAluc (Firefly luciferase) and phRL-CMV (Renilla luciferase). Normalized data for each cell population presented are the luminescence ratio *Firefly luc/Renilla luc*. Experiments were done three times in triplicate. Error bars represent the standard error of the mean and p < 0.05 was considered a significant variation (Mann- Whitney U test).

We performed Western blot analysis to confirm the RelB expression profile in 22Rv1 PCa cells (Figure 1A). Based on a lentiviral expression system, we derived three independent clonal populations (C1, C2 and C3) of 22Rv1 cells that efficiently overexpressed RelB at different levels (low, medium to high expression in C1, C2 and C3 clones respectively) (Figure 1A-B). Using this lentiviral system, we also derived two GFP control populations, one polyclonal population (MP) and one independent clonal population (clone), which did not express exogenous RelB (Figure 1A-B). By immunocytochemistry, RelB expression appeared homogenous in the C2-RelB and C3-RelB clonal populations but not in the C1-RelB clone where we observed many cells with a very weak staining (Figure 1B).We tested the functionality of exogenous RelB in 22Rv1 cells by assessing its sub-cellular distribution and its association with other NF-κB subunits. By immunohistochemical assays on cultured cells, RelB distributed into both cytoplasmic and nuclear cell compartments in C1, C2 and C3 populations thereby suggesting a constitutive activation of the alternative pathway (Figure 1B). As expected, RelB was not detected in 22Rv1 GFP control cells (Figure 1B). Western blot analyses of fractionated cell extracts confirmed that RelB was distributed in both cytoplasmic and nuclear compartments of 22Rv1-RelB clones (Figure 2A). The principal molecular partners of RelB, p100 and p52, were also localized in both cell compartments of 22Rv1-RelB clones C1, C2 and C3 and in the GFP control populations (Figure 2A). Conversely, we noted that the main NF-κB subunit of the classical pathway, p65, was only cytoplasmic (Figure 2A). Immunoprecipitation assays showed a physical interaction between RelB, and both p100 and p52 in the cytoplasm. In the nuclear compartment, only p52 appeared physically linked to RelB, thus formed RelB/p52 dimers (Figure 2B). The presence of RelB/p52 in the nuclear compartment suggested a constitutive activation of the alternative NF-κB pathway, whereas the absence of nuclear p65 indicates a lack of classical NF-κB pathway activation.We further measured NF-κB transcriptional activity by a luciferase gene reporter assay. We observed a significantly higher NF-κB transcriptional activity in the 22Rv1-RelB clones than in GFP control populations (Figure 2C). Since the classical NF-κB p65 subunit was not nuclear in 22Rv1 cells, our data suggest that the increased κB transactivation in RelB clones was exclusively due to the alternative NF-κB activity. Together, these observations illustrate that the exogenous RelB expression induced an efficient activation of the alternative NF-κB pathway in transduced 22Rv1 cells.

### RelB caused a lag the tumor growth of 22Rv1 cells in mice

**Figure 3:**
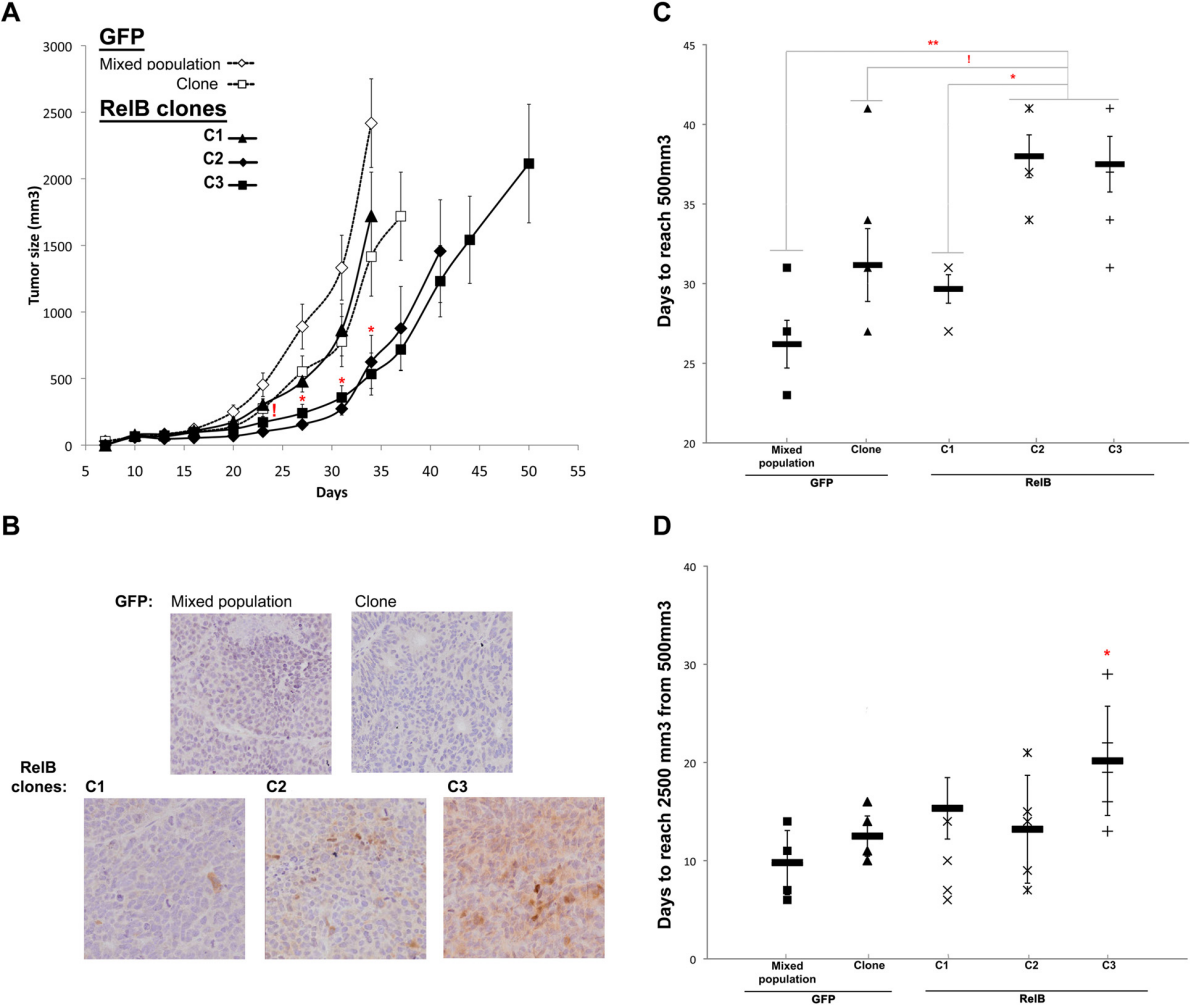
**RelB expression effect on 22Rv1 induced-tumor formation in a SCID mouse model.** Analysis of 22Rv1-induced tumor growth. 250,000 22Rv1 RelB or GFP cells suspended in matrigel solution (5 mg/mL) were injected subcutaneously in the flank of 6-weeks old SCID mice. For each 22Rv1 cell population, 6 mice were injected. **A)** Tumor size was graphically represented across time. For each group of mice, the last point of the curve corresponds to the time of the first mouse sacrifice. Error bars represent the standard error of the mean. Significant variation between C2- and C3-RelB mouse groups individually compared to each GFP control (MP and clone) and C1-RelB mouse groups is illustrated by (*) on graph whereas (!) represents a significant variation between C2 and C3 RelB mice individually compared to MP GFP control group (Kruskal-Wallis test). **B)** Immunohistochemical staining of RelB illustrating its expression status on harvested 22Rv1-induced tumors. **C)** Time for 22Rv1-induced tumors to reach 500 mm^3^. On graph, (*) illustrated a p < 0.05; (**) p < 0.001. **D**) Time for 22Rv1-induced tumors to grow from 500 mm^3^ to 2500 mm^3^. (*) illustrates p < 0.05. Error bars represent the standard error of the mean (Anova one-way test followed by a Tukey post-test).

To assess the impact of RelB on the tumorigenicity of 22Rv1 cells *in vivo,* we performed subcutaneous injections of 22Rv1-RelB clones or 22Rv1-GFP cells into SCID mice and monitored the rate of tumor growth (Figure 3). Our data showed that RelB expression in C2-RelB and C3-RelB cells increased significantly the time of tumor initiation compared to GFP-control cells. Indeed, at day 23 post-injection, we observed that the size of GFP control mixed population (MP) tumors reached 453.46 (±88.97) mm^3^ while the sizes of C2-RelB and C3-RelB tumors were 101.37 (±12.53) and 171.37 (±43.88) mm^3^, respectively (*P* < 0.05, Kruskal-wallis test). In contrast, we noted that C1-RelB tumors grew similarly to the GFP clone control tumors (Figure 3A).

To confirm the expression of RelB in the xenografts we performed histological analyses of harvested tumors. Immunohistochemical assays showed that RelB expression was maintained in C2-RelB and C3-RelB tumors (Figure 3B). C1-RelB tumors have only a few scattered cells expressing RelB and the overall RelB expression appeared very low as observed with *in vitro* cultured C1-RelB 22Rv1 cells (Figure 1). As expected, there was no RelB expression in the control GFP tumors (Figure 3B). RelB expression in C2- and C3-RelB tumors was respectively moderate and strong, as observed by immunoblot and immunocytochemical assays of cultured 22Rv1 clones (Figure 1).

The observed lag in tumor growth of C2-RelB and C3-RelB xenografts was mainly observable during the first phase of tumor formation (i.e. the first 23–34 days post-injection) corresponding to the tumor initiation phase (Figure 3A). Indeed, C2- and C3-RelB tumors reached a size of 500 mm^3^ in 38 (±1.34) and 37.5 (±1.74) days respectively, while endpoints (2500 mm^3^) were reached in 26.2 (±2.67) and 31.2(±1.5) days for the GFP control tumors (MP, clone), and in 29.7 (±0.9) days for C1-RelB xenografts (Figure 3C). The difference in the initial tumor growth rate between 22Rv1 RelB xenografts (C2 and C3) and MP GFP control was statistically significant (*P* < 0.001, Tukey’s test). Due to one extreme value in GFP clone group, the difference with C2- and C3-clones tumors was not significant (GFP clone/C2: p = 0.065; GFP clone/C3: p = 0.078). The statistically significant variation in 22Rv1-RelB tumors between C2 or C3 and C1 that weakly expressed RelB (Figure 3B) supported the role of RelB in the tumor growth rates (respective comparison C1/C2 and C1/C3; *P* = 0.019 and *P* = 0.021, Tukey’s test) (Figure 3C).

Once 22Rv1-induced tumors reached 500 mm^3^, their growth rates increased notably and did not vary significantly between different groups except for C3-RelB tumors (*P* < 0.05 compared to all others groups, Tukey’s test). Whereas C1-, C2- RelB clones and GFP control tumors took 9.8 to 15.3 additional days to reach 2500 mm^3^, C3-RelB tumors required 20.2 days (*P* < 0.05, Tukey’s test) (Figure 3D). Nevertheless, at the end, all 22Rv1 cells, including the C3-RelB clone, induced an important tumor growth (Figure 3A). This observation suggested that RelB expression did not inhibit the tumor growth despite the lag in the initial phase of 22Rv1 tumor formation. Together, these results suggest that the overexpression of RelB caused a lag in tumor initiation without affecting the overall tumor growth.

### RelB stimulated proliferation but inhibited the anchorage-independent growth of 22Rv1 cells

**Figure 4:**
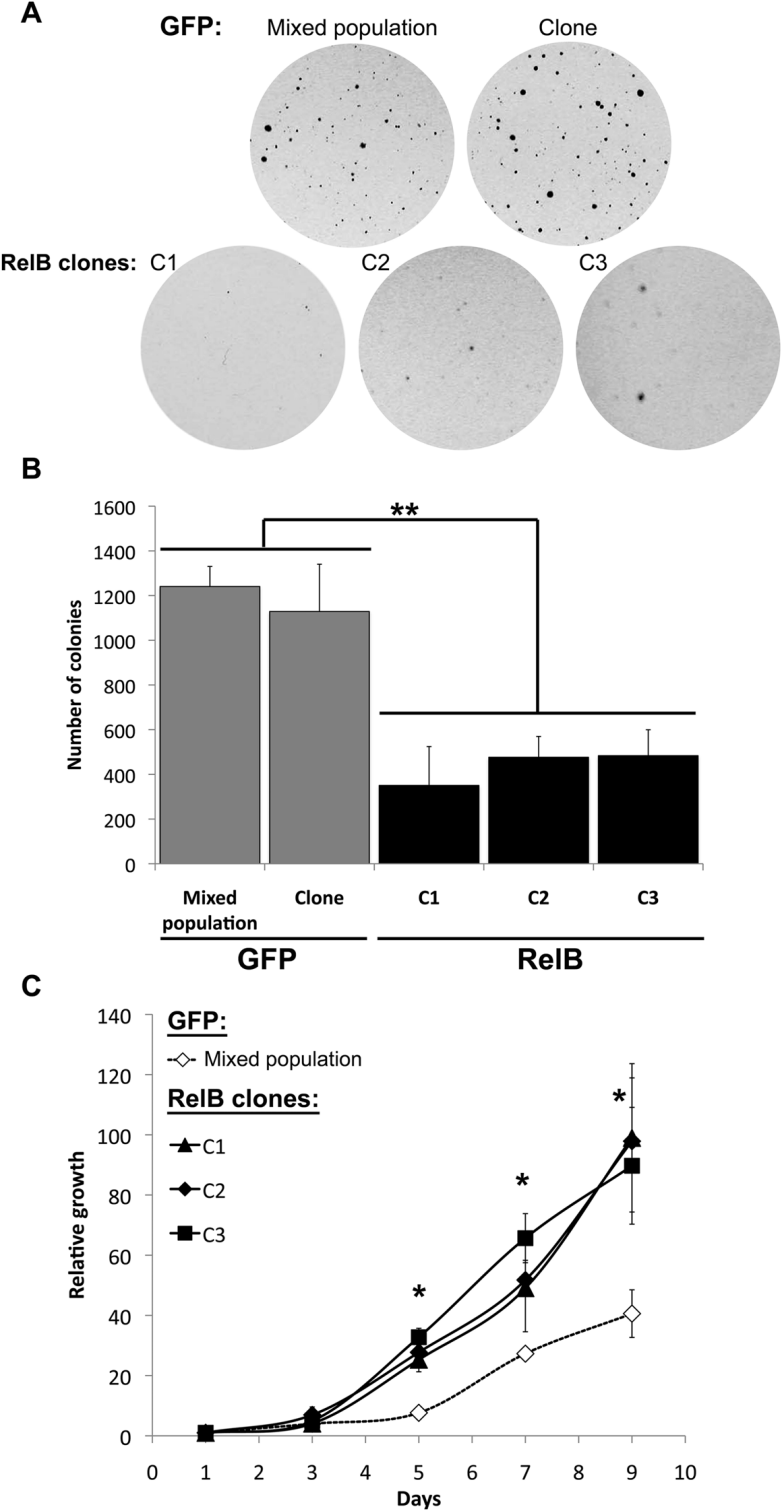
**Effect of RelB expression on cell proliferation and anchorage-independent cell growth of 22Rv1 cells. A)** Representative field of 22Rv1 colonies (macroscopic view) illustrating 22Rv1 RelB and GFP cells grown in soft agar. **B)** Soft agar growth assay. 22Rv1 cells were seeded in soft agar and incubated for 2 weeks. Colonies were stained with crystal violet. Graph represented the number of colonies in soft agar. Experiments were done three times in duplicate. Error bars represent the standard error of the mean and p < 0.05 was considered a significant variation (Mann- Whitney U test). **C)** Growth curves by cell counting. 22Rv1 RelB clones and GFP control cells were seeded at day 0. The first cell count was done at day 1 and after, cells were counted every 2 days until day 9 (10 days after cell seeding). The relative cell growth was obtained by normalizing data to the cell count at day 1. Experiments were done three times in duplicate. Error bars on the graph represent the standard error of the mean and we consider a p < 0.05 significant variation (Anova one-way followed by Tukey post-test).

The *in vivo* observations on tumor initiation and progression led us to explore different *in vitro* cell processes to better understand the impact of RelB in PCa cells. We first assessed the anchorage-independent cell growth of 22Rv1 cells and the effect of RelB expression in a soft agar assay. The 22Rv1-RelB clones formed an average of 2.3 to 3.4 fewer colonies than the GFP control cells (MP and clone), (p < 0.001, Mann–Whitney U test) (Figure 4A, B). Thus, RelB expression in 22Rv1 cells decreased their ability to grow independently to anchorage with extracellular matrix.Because the anchorage-independent cell growth is tightly related to cell proliferation, we also addressed the impact of RelB on proliferation rate of 22Rv1 cells cultured in monolayer. We performed a cell counting assay using the RelB clones (C1, C2 and C3) and GFP control cells. Surprisingly, from day 5 of cell culture until the end of the assay, we observed a significant increasing of cell proliferation of the three 22Rv1-RelB clones compared to GFP control cells (p < 0.05, Kruskal-Wallis test) (Figure 4C) indicating that RelB expression stimulated the proliferation of 22Rv1 cells in monolayer conditions. Altogether, these results suggest that the RelB-induced inhibition of anchorage-independent cell growth was not caused by an inhibition of the cell proliferation.

### RelB increased susceptibility of cells to undergo cell death

**Figure 5:**
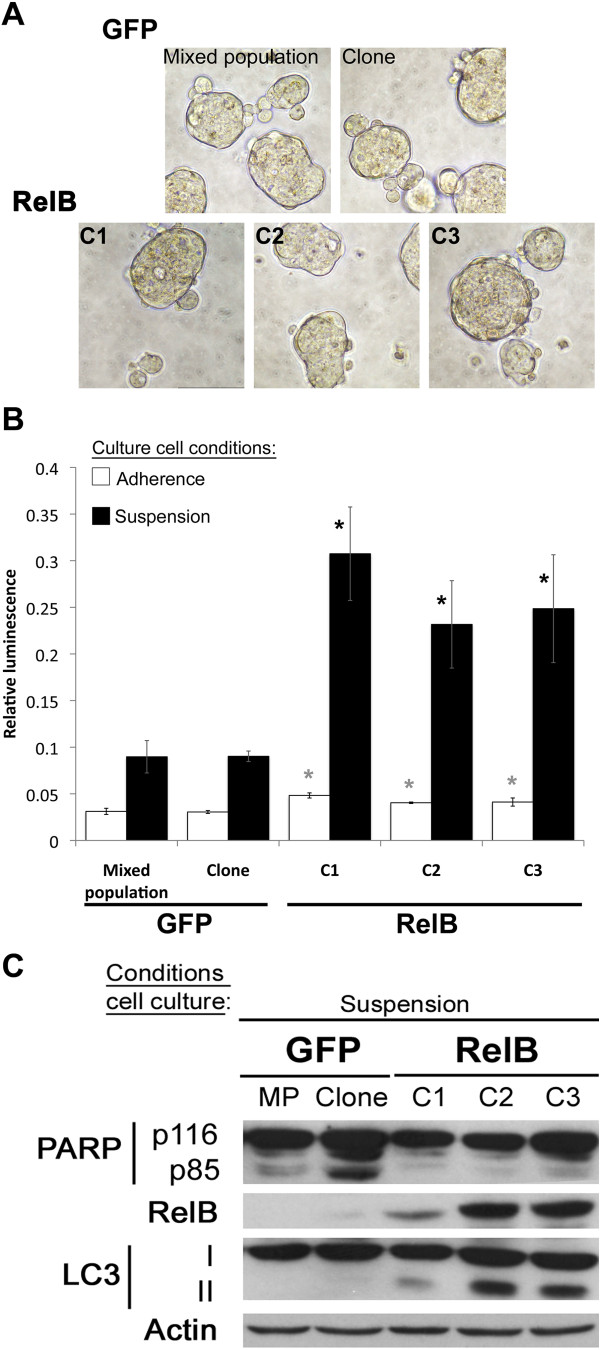
**RelB effects on cell death of 22Rv1 cells. A)** Illustration of 22Rv1-derived cells cultured in suspension on poly-HEMA pre-coated plates. Representative fields at 200X magnificence. **B)** Cell death evaluation in suspension cell culture of 22Rv1-derived cells. Cells were cultured 7 days in 96-well plates pre-coated or not with poly-HEMA. Cell death was evaluated by bioluminescence using the CytoTOX-Glo cytotoxicity assay. Data represent the ratio of *basal cell death/total cell death* after complete lysis using digitonin reagent. Cell death in suspension culture conditions are illustrated in black bars and adherence control cell culture conditions in empty bars. The variation was significant at p < 0.05 (Mann- Whitney U test). (*) illustrates significant variations with both GFP control populations. Error bars represent the standard error of the mean. **C)** Immunoblot analyses of cell death from whole protein extracts of 22Rv1-derived cells following 7-day suspension cell culture RelB status, PARP cleavage (apoptosis) and LC3-I to LC3-II conversion (autophagy) were evaluated. Actin was used as loading control.

The anchorage-independent cell growth is a cell process dependent on the ability to proliferate while resisting cell death induced by the loss of cell-cell and cell-extracellular matrix (ECM) interactions [26]. We thereby addressed whether RelB had an effect on cell death induced by the loss of anchorage to ECM, which might explain their reduced growth in soft agar (Figure 4). To do so, we cultured 22Rv1-derived cells in suspension (Figure 5A) to mimic anchorage-independent cell growth conditions and used adherent conditions as controls. Under adherent conditions, 22Rv1 RelB clones underwent slightly more cell death (0.040 – 0.048 ± 0.001 - 0.003) compared to GFP control cells (0.030 - 0.031 ± 0.001 - 0.004) (Figure 5B; white bars: *p* < 0.05, Mann-Whitney U test). Furthermore, under non-adherent conditions, we observed that RelB increased dramatically the rate of cell death by 2.5 - 3 fold in 22Rv1-RelB clones (0.232 – 0.307 ± 0.054 - 0.057) compared to the GFP control cell populations (0.089 – 0.090 ± 0.006 - 0.017) (Figure 5B; black bars: *p* < 0.05, Mann – Whitney U test).

It is known that in response to the loss of link with ECM, cells undergo caspase-dependent apoptosis, also known as anoikis [26,27]. To characterize the cell death mechanisms involved in our model, we first assessed PARP (Poly-ADP-Ribose polymerase) cleavage (from p116 to p85), a commonly used caspase-dependent apoptosis marker on 22Rv1 cells cultured in suspension. Under suspension cell culture conditions, we did not observe any p85 PARP fragment in 22Rv1-RelB clones comparatively to 22Rv1-GFP control cells where p85 PARP was detected (Figure 5C). The absence or very low level of PARP cleavage in 22Rv1-RelB cells indicated that, despite an increase cell death ratio (Figure 5B), 22Rv1-RelB clones underwent a low rate of apoptosis.

The loss of anchorage can also trigger compensatory mechanisms to overcome anoikis. Autophagy has been described as an alternative mechanism that can be triggered after matrix detachment of adherent cells [28,29]. We explored the possibility that RelB could regulate autophagy after loss of anchorage to ECM by analyzing the LC3 protein, whose conversion from cytoplasmic LC3-I to autophagosomal LC3-II is a specific marker of autophagy [30]. As shown in Figure 5C, under suspension cell culture conditions, we observed LC3-I conversion into LC3-II in 22Rv1-RelB clones when compared to GFP control cells indicating that expression of RelB sensitized 22Rv1 cells to autophagy. These observations also suggest that RelB could protect 22Rv1 cells against anchorage-dependent apoptosis by triggering autophagy. *In vitro,* the observed autophagy might ultimately induce 22Rv1 cell death due to the length of the assay without restoring adherent conditions (7 consecutive days under suspension culture conditions).

## Discussion

Our previous study regarding the expression profile of different NF-κB subunits in PCa tissues from patients illustrated the nuclear localization of alternative NF-κB subunits RelB and p52, thereby suggesting a potential activation of the alternative NF-κB pathway [13]. These results led us to further investigate the role of the alternative NF-κB pathway in PCa.

Different cell lines are currently used as models to study PCa and can be classified according to their response to androgen stimulation and their castration-resistance status. Previous studies on the NF-κB alternative pathway in PCa used the PC3 and LNCaP cell lines [14,15]. PC3 is a castration-resistant cell line with a high metastatic potential whereas LNCaP is an androgen-dependent non-metastatic cell line [31,32]. Both PC3 and LNCaP have a constitutive NF-κB activity. Indeed, in PC3 cells classical and alternative NF-κB pathways are strongly and constitutively activated [25]. In contrast, in LNCaP cell line the classical NF-κB pathway is moderately active compared to PC3, whereas the alternative NF-κB activity is low, possibly due to the low levels of the alternative NF-κB subunit RelB, which can nonetheless be induced by a TNF-α stimulation [24]. Despite the fact that PC3 and LNCaP cell lines are currently used to study the functions of the NF-κB pathways in aggressive or non-aggressive PCa context, the constitutive presence of classical NF-κB activity could influence any interpretation on the effect of alternative NF-κB pathway in these cell lines. Just like the most differentiated prostate tumors derived LNCaP cells, 22Rv1 can respond to androgen stimulation (androgen-sensitive). The 22Rv1 cells can also grow in hormone-depleted conditions as the castration-resistant PC3 cells [33]. Like LNCaP cells, the 22Rv1 cell line have no basal alternative NF-κB activity, due to very low basal level of RelB expression. However, 22Rv1 have also the particularity to lack the classical NF-κB pathway as opposed to LNCaP cells [24,34]. These last characteristics make 22Rv1 cell line a particularly interesting model to study the alternative NF-κB pathway, with no interference of the classical NF-κB activity found in other common PCa cell lines. Therefore, in the present study we used the 22Rv1 cell line to specifically analyze the alternative NF-κB pathway and to define its role in tumorigenesis of PCa.

Our *in vivo* experiments with RelB transduced 22Rv1 PCa cells demonstrated that RelB expression caused a lag in tumor initiation, but only weakly affected the tumor growth rate once tumors had reach a volume of 500-mm^3^. *In vitro* results showed that RelB expression decreased the anchorage-independent growth of 22Rv1 cells, which correlated with the lag of tumor initiation of RelB-expressing xenografts. Indeed, cells lose cell-matrix attachment when they are trypsinized and suspended in a semi-solid environment at the time of injection, a condition that could mimic the stress cells undergo in the *in vitro* soft agar assay. In accordance with these results, we observed that in prolonged anchorage-independent culture conditions RelB is associated with induction of cell death and autophagy.

The lag in the tumor initiation observed with RelB-22Rv1 cells is in contrast with previous studies reporting that LNCaP xenografts expressing RelB grow faster than the parental LNCaP cells [14,15]. Furthermore, LNCaP cells overexpressing RelB have an increased ability to grow in anchorage-free conditions [8]. These contrasting results suggest that the effect of RelB is highly contextual, and also suggest the interplay of several critical regulators of cell growth and death in PCa cell lines. Considering the different molecular phenotypes of both cell lines, the observed differences with 22Rv1 cells brings supplemental information as to the impact of RelB on PCa cell biology. Several molecular mechanisms are involved in the resistance to anoikis of tumor cells, including survival signaling driven by PI3K and NF-κB pathways, the expression of anti-apoptotic proteins such as Bcl-2, and the hyperactivation of tyrosine kinase receptors such as EGFR and ErbB2 [26]. LNCaP cells constitutively present a strong basal PI3K activity, as observed by the phosphorylation of Akt compared to 22Rv1 cells [35]. The overexpression of RelB in these contexts may also promote distinct pro- or anti-survival pathways that make LNCaP and 22Rv1 cells behave differently during tumorigenesis.

To date, several reports have described how autophagy can impact NF-κB signaling by inducing the degradation of IKK (Inhibitor κB kinases) and NIK (NF-κB-induced kinase), upstream regulators of both the classical and alternative NF-κB pathways [36]. Furthermore, it is also reported that the classical NF-κB pathway (p65 subunit) can regulate autophagy through the transcription of target-genes such as BECN1, coding for Beclin-1, an important protein involved in autophagy signaling [37-39]. This is the first study to show a role of the alternative NF-κB pathway in autophagy.

**Figure 6:**
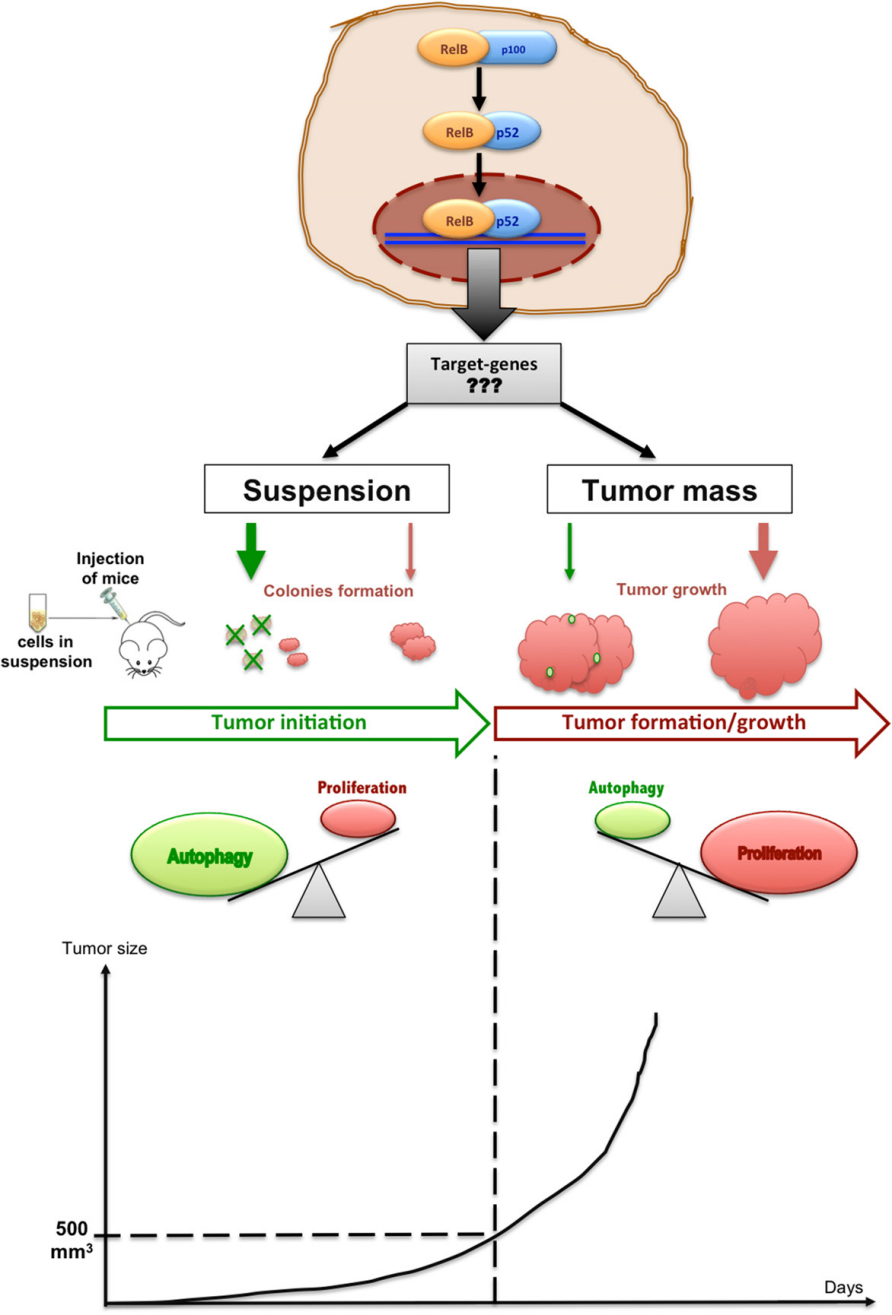
**Model of RelB influence in 22Rv1 cells in xenograft assays.** In 22Rv1 cells, exogenous RelB induces the activation of alternative NF-κB pathway, resulting in undetermined target-genes and specific cell responses. The tumor growth initiation *in vivo*, cells are suspended in semi-solid media, mimicking anchorage free conditions that trigger autophagy in the RelB expressing cells (green lined pink colonies). At this time there is a low proportion of proliferating cells (red colonies) and the balance *autophagy/proliferation* weighs in favor of autophagy. Triggered autophagy then protects 22Rv1-RelB cells from stress induced by suspension conditions until they recover optimal growth conditions once they are anchored within mouse ECM. The colonies continue to grow and form the tumor mass (until 500 mm^3^ of tumor size) where adherence conditions predominate. At this time the balance *autophagy/proliferation* weighs in favor of proliferation resulting in increase of tumor growth rate.

Many studies have demonstrated a link between autophagy and cancer [40]. Based on its dual roles in survival and cell death, autophagy’s impact in tumorigenesis is still difficult to establish and may depend on the cancer cell microenvironment [40-42]. For adherent epithelial cells such as 22Rv1, the loss of anchorage with ECM constitutes to a loss of growth signals transduced through integrin/src signaling, inducing a stress able to trigger autophagy [43,44]. Normally, the loss of cell anchorage with ECM induces anoikis [27,43]. We observed that the parental 22Rv1 cells appear to be resistant to the apoptotic cell death associated with anoikis, as suggested by their robust growth in the soft agar assay (Figure 4). The failure of RelB overexpressing 22Rv1 cells to efficiently grow in soft agar may reflect a cell protective response where autophagy is induced to promote cell survival but at the expense of cell growth. We propose the model in Figure 6 to explain dual RelB roles in tumorignesis of PCa in mice. By inducing autophagy in 22Rv1 cells, RelB provides an alternative protective process against anchorage free conditions allowing cells to integrate mouse ECM, until condition are more favorable for cells to proliferate. By transposing this model to the human PCa context, RelB could be involved in the metastatic process and protect cells during their transit towards metastatic sites. However, further experiments are needed to determine if RelB regulate an autophagy flux and leads of the cell death observed in RelB expressing clones.

The current study highlights the tumorigenic potential of the alternative NF-κB pathway in PCa cells. While previous reports show that RelB could intervene in potential tumorigenic cell processes as proliferation, invasion and resistance to several anti-cancer therapies, this is the first study to show that the alternative NF-κB pathway can induce autophagy in an anchorage-free assay. This new function associated with the alternative NF-κB signaling brings additional knowledge about the tumorigenic mechanisms involved in progression of PCa. Supplemental studies are necessary to define the molecular actors involved in the autophagy regulated by the alternative NF-κB pathway.

## Competing interest

The authors declare that they have no competing interests.

## Authors’ contributions

IL designed the study, carried out all cell biology experiments and drafted the manuscript. AP produced the plasmid constructs. JL carried out the lentiviral infection. AP and JL had an equivalent contribution in the current study. ND carried out the selection of clonal cell populations. POG participated in the design of the study. CLP participated to draft the manuscript. FD and AMM conceived of the study and participated in its coordination and helped to draft the manuscript. All authors read and approved the final manuscript.
